# Recalling Memories of Life’s Challenges with Purpose and Redemption: Resources to Foster Resilience?

**DOI:** 10.1093/geroni/igab046.3277

**Published:** 2021-12-17

**Authors:** Shubam Sharma, Joshua Perlin, Susan Bluck

**Affiliations:** 1 Kennesaw State University , Kennesaw, Georgia, United States; 2 University of Florida, Gainesville, Florida, United States; 3 University of Florida, University of Florida, Florida, United States

## Abstract

Unique life challenges occur across life phases, including later life. Life story research suggests that the way challenges are narrated has consequences for multiple domains of well-being. Two factors for positively reframing challenges are one’s sense of purpose in life (Windsor et al., 2015) and redemption (McAdams et al., 2001). This study used moderated-mediation analyses to investigate whether: 1) challenge relates to psychosocial and cognitive well-being, differentially by age, and 2) narrating with greater purpose and redemption ameliorates effects of challenges on well-being, by age. Participants (N = 99 young, 88 older adults) rated self-disruption of challenging events from their lives (IV1) and reported number of recent life challenges experienced (IV2). Eudaimonic well-being (DV1) and cognitive well-being (DV2) were assessed. Purpose (M1) and redemption (M2) were reliably content-analyzed from participants’ narratives of autobiographical challenges (e.g., illness, loss of other). For Aim 1, young and older adults who experienced more challenges showed lower eudaimonic well-being but higher cognitive well-being. Perceived self-disruption was unrelated to well-being. For Aim 2, results showed that how individuals narrate (i.e., with purpose, with redemption) the challenges that have occurred in their lives mediates effects of challenge. Specifically, exhibiting a sense of purpose mediated the relation between perceived self-disruption and cognitive well-being. Redemption buffered negative effects of both self-disruption and number of challenges on eudaimonic well-being. Mediation results held regardless of age. Findings suggest purpose and redemption are two narrative mechanisms that act as psychological resources to support well-being in the face of life’s inevitable challenges.

